# Biocompatible neem gum-modified polyvinyl alcohol composite as dielectric material for flexible energy devices

**DOI:** 10.1016/j.heliyon.2024.e28379

**Published:** 2024-03-20

**Authors:** Kiruthika Parangusan, Venkat Subramaniam, Anandha babu, P. Sundara venkatesh, S. Vijayalakshmi, Deepalekshmi Ponnamma

**Affiliations:** a Department of Electronics, PSG College of Arts and Science, Coimbatore, Tamilnadu, India; b Department of Physics, Bannari Amman Institute of Technology, Sathyamangalam, Tamilnadu, India; c Department of Physiology, Saveetha Dental College and Hospitals, Saveetha Institute of Medical and Technical Sciences, Saveetha University, Chennai, 600077, Tamilnadu, India; d Department of Physics, Sri. S. Ramasamy Naidu Memorial College, Sattur, 626203, Tamilnadu, India; e Materials Science and Technology Program, Department of Mathematics, Statistics and Physics, College of Arts and Sciences, Qatar University, 2713, Doha, Qatar

**Keywords:** Polymer blends, Neem gum, Composite, Dielectric properties, Bio-electrode, Energy storage

## Abstract

In our pursuit of a flexible energy storage solution, we have developed biocompatible (bc)-NG/PVA composite polymers by combining neem tree gum (NG) with polyvinyl alcohol (PVA). This innovative bio-inspired approach harnesses NG's unique properties for both the bio-electrolyte and bio-electrode components. The resulting bc-NG/PVA composites exhibit superior dielectric strength and versatility, surpassing traditional inorganic ceramic dielectrics in advanced electronics and pulsed power systems. Our study investigates the dielectric characteristics, conductivities, electric modulus, and impedance parameters of Pure PVA and NG-doped PVA composites. Adding 5 % NG to PVA significantly boosts its conductivity from 10^−8^ S cm^−1^ to 10^−4^ S cm^−1^, while the dielectric constant of PVA/5 % NG composite jumps to 104.5 compared to pure PVA. These improvements position the composite films of 5 % NG added PVA as promising materials for diverse applications. The heightened performance of these NG-blended PVA composite materials underscores their potential as a valuable resource for flexible energy storage solutions.

## Highlights

• Neem gum enhances PVA dielectric properties.
• Structural analysis reveals improved characteristics.
• Promising for flexible energy storage applications.

## 1 Introduction

The global population and the utilization of fossil fuels are expanding rapidly. In this context, the development of renewable energy technologies is crucial for future energy generation, storage, and utilization [[1], [2], [3]]. Dielectric materials exhibit outstanding features that make them valuable for electronics due to their adaptability, versatility, and eco-friendliness [4]. Compared to ceramics, polymers offer greater flexibility, processability, and lightweight properties, overcoming several limitations associated with ceramics such as embrittlement, processing challenges, and low stability [5]. Numerous researchers have successfully developed super-absorbent polymeric hydrogels with the capability to store a significant amount of water and biological fluids within their cross-linked polymeric network structure. In recent years, there has been an increased interest in the biocompatibility and pH sensitivity of these hydrogels [6,7].

The advanced manufacturing methods associated with these electrolytes indicate that they may be suitable for powering future environmentally sensitive energy generation systems. The combination of solid materials and polymer electrolytes has recently gained attention due to its benefits in terms of safety and environmental friendliness. For example, Lee et al. reported on clay composites with elevated ionic conductivities, mechanical robustness, and flexibility [8]. Yan et al. reported on a disposable bacterial cellulose-supported quasi-solid electrolyte [9], which exhibits better stability with temperature variations and a higher decomposition rate. Additionally, Wang et al. developed a high-performance lithium battery [10] by incorporating a polymer-laden lignin electrolyte. Goma et al. developed a graphene-doped polymer-in-polymer composite (70 % PVP/30 % PVA) with remarkable optical and electrical properties, making it an outstanding candidate for applications in optoelectronics, laser limiters, optical filters, and biomedical lasers [11]. Badri et al. demonstrated that ZnO and GO-substituted PVA, produced using the traditional casting procedure, could serve as solid polymer electrolytes in optoelectronic applications [12]. Hasim et al. developed various nanostructures, including PVA/SnO_2_/SiC, PVA-TiN-SiO_2_, PS/SiO_2_/SrTiO_3_, PS/SiC/CeO_2_, PMMA-doped SiC/Y_2_O_3_, PMMA/CoFe_2_O_4_/ZnCoFe_2_O_4_, and PVA/CuO films. These materials exhibit improved dielectric constant (*ε*′) and electrical conductivity (σ_AC_), making them suitable for potential and energy storage applications [[13], [14], [15], [16], [17], [18], [19]]. Similarly, a variety of novel polymer blends, including PVA/PVP/CMC-ZnO hybrid nanocomposite, PVA/PVP/CMC ternary polymer blend, hybrid nanofillers (Gold quantum dots and Copper nanoparticles) on the PEO/CMC blend, and PVA/SA blend with Ag/Se nanofillers, were created using the solution casting method. These blends demonstrate remarkable dielectric properties for use in various energy-related applications [[20], [21], [22], [23], [24], [25]].

However, there are disadvantages to using naturally generated polymers, including the possibility of microbial contamination, uncontrollably high viscosity, and changes in viscosity over time. Various tactics are employed to modify the properties of the polymer in an effort to strengthen and maintain its homogeneity. Grafting, mixing, and curing are common methods for hydrogel modification. Cross-linked hydrogels find wide application in pharmaceuticals, tissue engineering, and wound care. Addition of NH_2_, COOH, OH, SO_3_, and CONH_2_ groups enhances water absorption in polymers. Graft copolymers aid soil moisture retention, benefiting agriculture, reducing plant mortality, conserving water, and improving soil fertility, especially in arid regions [[26], [27], [28]]. The PMMA/PEG films and Si_3_N_4_ NPs doped PMMA/PEG were manufactured using a casting process, and the findings of dielectric properties showed that the dielectric parameters of PMMA/PEG increased as the Si_3_N_4_ NPs concentration increased. Furthermore, Si_3_N_4_/PEG/PMMA nanostructures have excellent optical properties, making them potentially useful in prospective optics and electronics applications due to their low cost, flexibility, and good physical and chemical properties compared to other nanomaterials [29,30].

Neem gum (NG), a naturally occurring water-soluble polysaccharide, is derived from the exudates of Azadirachta indica, a member of the Meliaceous family [31]. NG has been explored in pharmaceutical dosage forms, serving as a binder and excipient [32,33]. Gums extracted from various trees, including Neem, offer non-hazardous, renewable, biocompatible, and cost-effective electrolyte options, presenting several advantages [34,35]. Therefore, the addition of NG to polyvinyl alcohol (PVA) results in unique modifications, offering increased resistance to microbial growth, enhanced mechanical strength, improved biodegradability, and barrier qualities against gases, moisture, and UV light. NG's bioactive release capacity and rheological properties make it suitable for regulated delivery in medicine and agriculture. Additionally, its promotion of substrate adherence enhances coated or bonded product performance. Overall, incorporating NG into PVA presents a promising approach for developing environmentally sustainable materials with diverse functionalities tailored to specific applications.

In this study, we developed a PVA/NG polymer composite for flexible energy applications by blending naturally accessible NG with PVA. Comprehensive analyses using XRD, FTIR, SEM, impedance analyzer, and dielectric investigations demonstrate that the NG/PVA composite polymer exhibits favorable physicochemical and dielectric properties. The dc conductivity of the PVA blended with 5% NG ranged from 7.19 × 10^8^ to 5.49 × 10^7^ S/cm. Variations in the NG concentration within the host matrix lead to notable improvements in dielectric characteristics and relaxation factors, indicating significant changes in polymer supportive chain segmental movement.

## 2 Experimental section

### 2.1 Collection and purification of neem (Azadirachta Indicia) gum

Neem (Azadirachta Indica) dried gum was obtained by puncturing the bark of locally grown Azadirachta indica trees. The moisture was eliminated by desiccating NG, exposing it to direct sunlight continuously for 7 days. Subsequently, the gum was finely powdered using a mortar and pestle. Three grams of NG were dissolved in 50 ml of deionized water with constant stirring at room temperature for 4–6 h. The solution was filtered using Whatman filter paper to remove any impurities present in the NG. The resulting NG solution (density of 0.81 × 10^−5^ mol cm^−3^) was stored at room temperature for further experimental processes.

### 2.2 Synthesis of PVA/NG composites

**Fig. 1:**
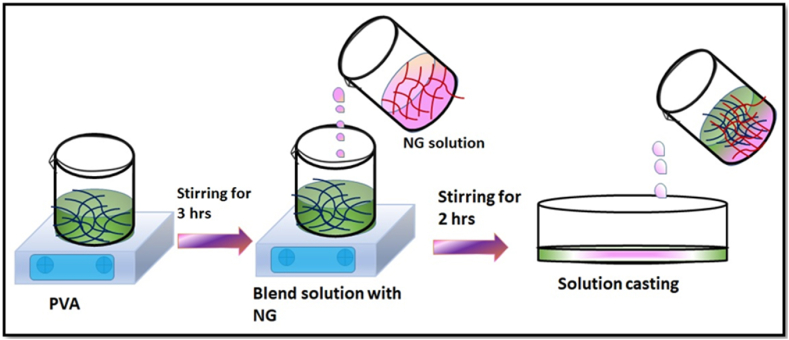
Experimental method of preparing PVA/NG composite.

The solution casting technique was employed to fabricate the blend polymer consisting of PVA and NG at different ratios. In this method, double-distilled water served as the solvent for preparing a biopolymer membrane. Initially, a particular concentration (2g) of PVA solution was dispersed in 20 ml of distilled water using magnetic stirring (400 rpm) for 5 h at 70 °C. Bath sonication was then employed to incorporate weight fractions (1, 3, and 5 wt%) into the same solvent mixture. These solutions were mixed and stirred overnight. The resulting mixtures of pure biopolymer (PVA) and polymer composites were allowed to dry in a hot air oven at 60 °C. Fig. 1 illustrates the experimental procedure for preparing the PVA/NG composite.

### 2.3 Characterization of PVA/NG composites

The structure of the composite films was analyzed using an X-ray diffractometer with a wavelength source of 1.540 Å, in steps of 0.05°, covering the range from 10 to 60°. The chemical composition of the prepared samples was assessed using a Shimadzu IR Tracer 100 spectrometer within the range of 4000–400 cm^−1^. The surface morphology of the prepared pure PVA and its composite films was examined using scanning electron microscopy (SEM) (Model: ZEISS-EVO 18 Research, Japan). The response of the prepared samples was analyzed using a Hioki 3532-50 LCR Hi-tester with an applied voltage at a frequency range from 42 Hz to 1 MHz. The polymer film was placed between two silver electrodes in an LCR Hi-tester, and Z-view fitting software was utilized to determine the overall resistances of the prepared samples.

## 3 Results and discussion

### 3.1 Structural and morphological properties of PVA/NG composites

**Fig. 2:**
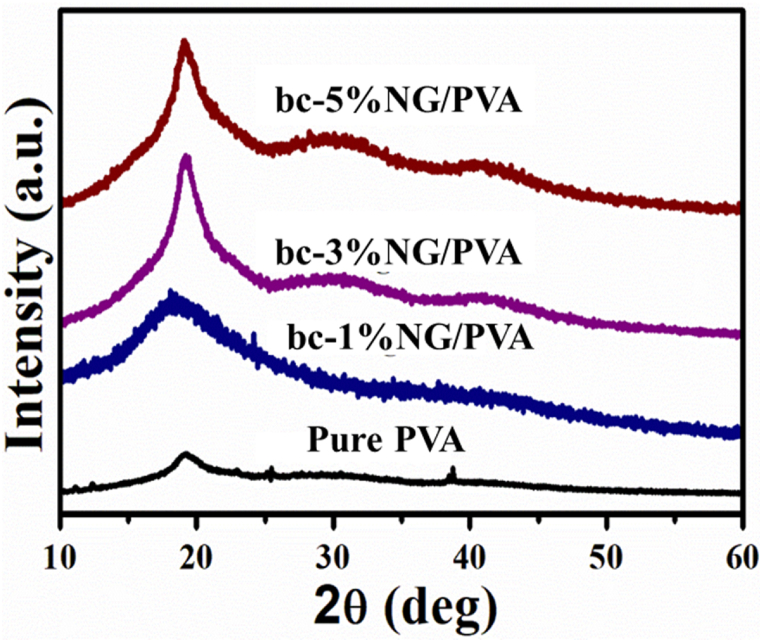
XRD patterns of PVA and bc-NG/PVA composites.

The structural and morphological characteristics of the PVA/NG composites were investigated to understand the impact of NG on the polymer matrix. Fig. 2 illustrates the XRD pattern of pure PVA and (1%, 3%, and 5%) NG -added biocompatible (bc)- NG/PVA composite samples. The broad peak observed at nearly 2θ = 19° corresponds to PVA's semi-crystalline property [36]. The addition of NG has increased the intensity of the peak, contributing to the enhancement of the crystallinity of the polymer composite [37].

Crucial for a polymer to exhibit dielectric properties are crystallinity and planar orientation. The addition of NG improves interfacial interactions, inducing crystallinity in PVA, and is expected to enhance the dielectric properties, as reported in the literature [38,39]. According to Kolanthai and Bodkhe [40,41] et al. found that adding nanofillers increased the crystallinity of the polymer matrix, which contributed to the better piezoelectric properties of PVDF [[42], [43], [44]]. Various factors, including preparation methods and the presence of NG within the polymer, are responsible for the higher crystallinity observed in the PVA composites.

**Fig. 3:**
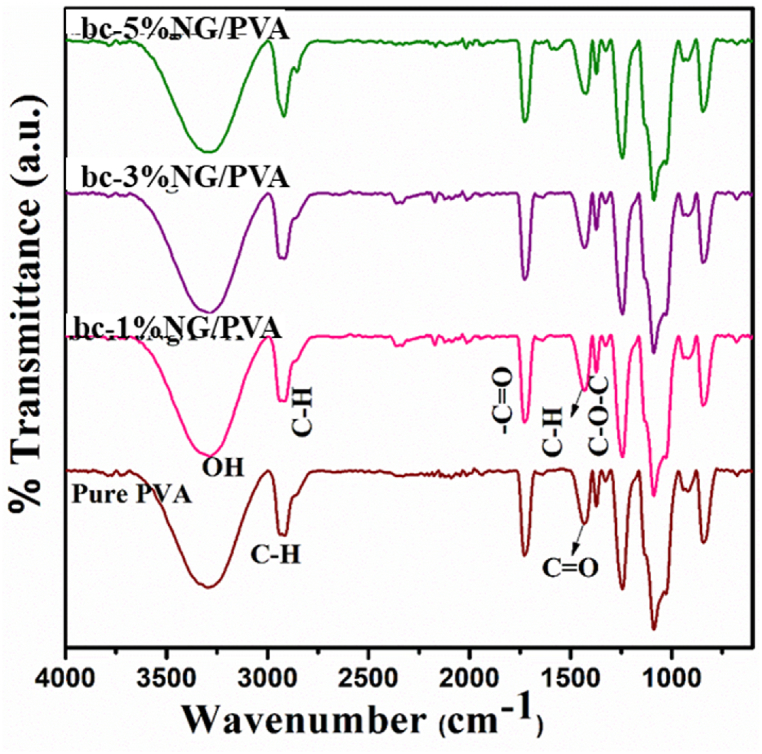
FTIR spectra of pure PVA and bc-NG/PVA composites.

Fig. 3 displays the FTIR spectra of pure PVA and (1%, 3%, and 5%) NG added bc-NG/PVA samples. In the FTIR spectrum of pure PVA, a broad peak at 3410 cm^−1^ signifies –OH stretching, indicative of intermolecular and intramolecular hydrogen bonds. A peak near 2960 cm^−1^ results from -C-H stretching of -C-*O*-CH_3_. The appearance of a peak at 1728 cm^−1^ is attributed to stretching vibrations of –C=O in the polymer.

In the FTIR spectrum of NG incorporated with the PVA matrix, bands at 3406 cm^−1^ indicate –OH stretching vibrations in the gum polysaccharide [45]. Peaks at 2931 cm^−1^ and 2121 cm^−1^ arise from the -C-H stretching mode of –CH_3_ groups in the gum and the overtones of -C-O stretching vibrations. Peaks at 1738 cm^−1^ and 1627 cm^−1^ correspond to C=O stretching vibrations of carboxylic acid and amide in the gum. Absorption peaks at 1421 cm^−1^ are due to C–H deformation of methylene groups in the gum, while those at 1248 cm^−1^ are due to C–*O*–C asymmetric stretching vibrations.

**Fig. 4:**
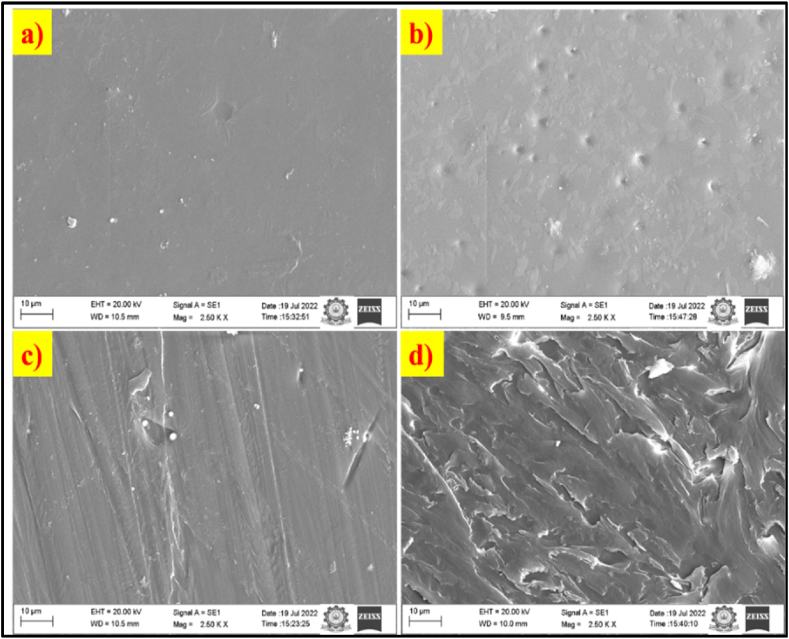
SEM Analysis of Pure PVA and bc-NG/PVA composites.

SEM is an effective method for investigating material morphology. In Fig. 4a, the SEM image of Pure PVA shows a homogeneous and consistent surface. The introduction of NG into pure PVA alters the surface morphology, as depicted in Fig. 4b-d. Significant changes occur with increasing weight percentage of NG in pure PVA. The 5% NG addition (Fig. 4d) shows increased surface change distribution, confirming interfacial contact between pure PVA and NG. The observed nanostructures in PVA/NG composites indicate the presence of different NG contents, enhancing ionic mobility, ion dissociation, and improving the mechanical strength of the polymer. The addition of 5% NG content may alter the surface properties of PVA, such as surface roughness, hydrophobicity/hydrophilicity, or surface charge, depending on specific application requirements. The strong interfacial bond between NG and the polymer matrix contributes to reinforced dielectric properties in these composites.

The addition of NG may chemically interact with the PVA matrix, forming stronger bonds at the interface between the NG particles and the PVA polymer chains. This interaction can lead to improved adhesion between the modified surface and other materials. Additionally, NG nanoparticles could act as crosslinking agents, forming additional bonds within the PVA matrix. This crosslinking can increase the overall strength and stability of the material, thereby improving bond strength.

### 3.2 Dielectric properties of the composite films

The complex conductivity σ*(ɷ) comprises of the real and imaginary part as given in Equation (1):

(1) $$\sigma^{*}\left(\omega \right)=\sigma^{\prime }\left(\omega \right)+j\sigma^{\prime \prime }\left(\omega \right)=j\omega \varepsilon_{0\;}\varepsilon^{*}\left(\omega \right)=\omega \varepsilon_{0}\;\varepsilon^{\prime \prime }+j\omega \varepsilon_{0}\varepsilon^{\prime }$$

Where σ′ the real part of conductivity and σ'' is the imaginary part of complex conductivity, ω is the angular frequency, ε_o_ (=8.854 × 10^−12^ F/m) is the dielectric constant of the free space.

**Fig. 5:**
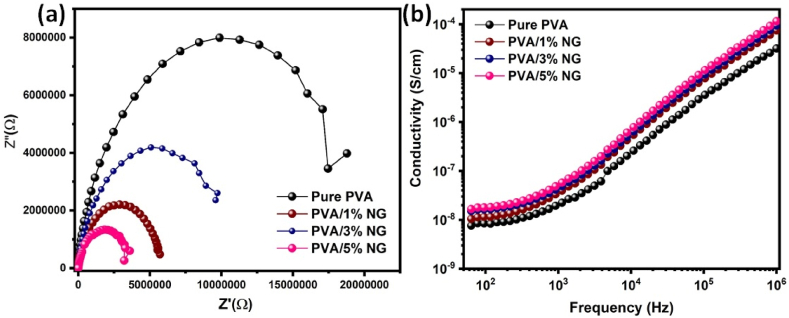
a) Nyquist curve derived from EIS findings and b) Variation of ac conductivity with frequency for pure and different NG (1, 3 and 5%) added PVA composites films.

Fig. 5a-b illustrates the Nyquist curve derived from EIS findings and differences in AC conductivity for composites with varying NG concentration levels and a pure PVA sample at different frequencies. As the neem content increases, the AC conductivity rises until reaching a 5% weight concentration. Beyond this concentration, conductivity decreases, primarily due to the combination of low-surface-area components forming larger structures [46]. Typically, the addition of NG causes an impedance modulation, which is frequently characterized by a drop in impedance magnitude due to enhanced interconnectivity among conductive parts within the composite structure, as shown in Fig. 5a. This improvement was noticeable, as 5% NG increased PVA conductivity from 10^−8^ S cm^−1^ to 10^−4^ S cm^−1^, outperforming all NG contents and pure PVA. This indicates that NG enhances conductivity or facilitates relaxing phenomena.

Furthermore, the addition of NG influences the frequency dependency of AC conductivity as shown in Fig. 5b, with composite films exhibiting lower frequency dependency. This results in a more stable conductivity profile across different frequencies, with possible conductivity improvements most noticeable at lower frequencies. The increased film conductivity of 104.5 achieved for PVA/5% NG composite compared to pure PVA is attributed to the formation of complexes in the composite samples and the presence of polar functional groups [47,48].

**Fig. 6:**
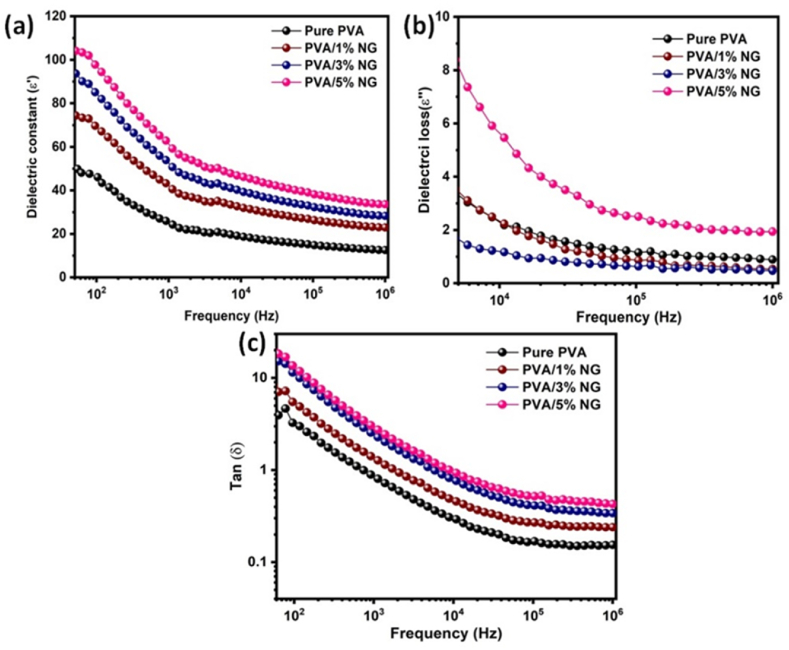
Frequency dependence of a) real dielectric constant, b) dielectric loss (*ε*″) and c) dielectric loss factor for Pure PVA and PVA with (1, 3 and 5%) added NG polymer composite films.

Fig. 6 depicts the frequency dependence of the real and imaginary parts of the dielectric constant. The dielectric constant is higher at lower frequencies, and the reverse effect is observed at higher frequencies, attributed to Maxwell-Wagner polarization predominantly caused by conductor-insulator interactions, indicating interfacial polarization. In the low-frequency range, space charges have sufficient time to respond to an applied electric field. In the higher-frequency range, alterations in the applied electric field occur too rapidly for space charges to compensate, and polarization cannot occur. PVA with 5% biocompatible NG content exhibits the highest dielectric constant, suggesting percolation. The dielectric constant tends to decrease above this percolation threshold [[49], [50], [51]].

Fig. 6b illustrates the decrease in dielectric loss (ɛ′′) as frequency increases. At higher frequencies, the reduction in dielectric loss is attributed to the diminished accumulation of polarization-induced charges due to the difficulty in generating space charges under the applied electric field [52]. Fig. 6c demonstrates the frequency-dependent evolution of the dielectric loss factor for all films. The graph indicates a decrease in the loss tangent as frequency rises. All things considered, the addition of 5% NG to PVA polymer composite films modulates these dielectric characteristics, which are frequently shown as shifts in peak values and changes in frequency response. In particular, there could be fluctuations in the actual dielectric constant as a function of frequency, and the presence of NG could result in increases or decreases in specific frequency ranges. NG incorporation also tends to affect the dielectric loss (*ε*″), as demonstrated by changes in peak values and frequency-dependent behavior. Furthermore, the addition of NG may change the dielectric loss factor, which is the ratio of dielectric loss to the true dielectric constant and reflects changes in the material's energy dissipation properties.

**Fig. 7:**
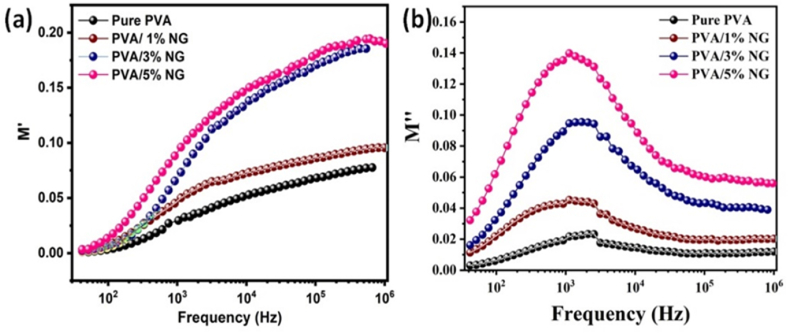
Frequency-dependent real part M′ and loss part M″ for pure and different NG added (1, 3 and 5%) added PVA composite films.

The electric modulus M* (ω) = 1/ε*(ω) can determine the complex permittivity ε*(ω) of the composite dielectric material. After accounting for impurities, electrode-dielectric interaction, and electrode polarization effects, these spectra validate the bulk response [53]. Fig. 7 displays the real M′ and imaginary M″ sections of the electric modulus calculated using permittivity profiles for pure PVA and various NG-added PVA composite polymers. The M′ value increases rapidly at high frequencies, indicating the dominance of non-Debye type relaxation behavior, particularly the Maxwell-Wagner-Sillars (MWS) relaxation mechanism. The widening peak at higher frequencies suggests the presence of PVA chain segment motions (-relaxation) in the studied composites [54,55]. The enhanced dielectric properties observed in the PVA/NG composite films, especially at a 5% NG concentration, can be linked to the improved structural features and increased interfacial contact between PVA and NG. The favorable dielectric strengths at higher frequencies make these composites suitable for applications in advanced electronics and energy storage devices.

## 4 Conclusion

The solution casting method was utilized to create PVA polymer composites with varying concentrations of NG. XRD patterns confirmed the successful fabrication of blended composites, while FTIR spectra revealed the interaction between NG and the polymer, resulting in complex formation through hydrogen bonding. The addition of NG significantly improved both electrical and dielectric properties, with the most notable enhancement observed at a 5 wt% NG concentration compared to pure PVA and other composite formulations. This enhancement was evident as NG boosted PVA conductivity from 10^−8^ S cm^−1^ to 10^−4^ S cm^−1^, while the dielectric constant of PVA/5% NG composite rose to 104.5 compared to pure PVA. At higher frequencies, the composites displayed favorable dielectric strengths, indicating potential applications across various devices. The incorporation of NG into the PVA matrix not only enhanced structural features but also showcased improved dielectric capabilities, thus rendering these composites promising for a wide array of device applications.

## Data availability statement

Data will be made available on request.

## CRediT authorship contribution statement

**Kiruthika Parangusan:** Methodology, Investigation, Formal analysis. **Venkat Subramaniam:** Resources, Investigation. **Anandha Babu:** Investigation. **P. Sundara Venkatesh:** Investigation. **S. Vijayalakshmi:** Visualization, Validation. **Deepalekshmi Ponnamma:** Writing – review & editing.

## Declaration of competing interest

The authors declare that they have no known competing financial interests or personal relationships that could have appeared to influence the work reported in this paper.
